# Hepatitis C Virus in the US Military Retiree Population: To Screen, or Not to Screen?

**DOI:** 10.14740/jocmr2233w

**Published:** 2015-08-23

**Authors:** Christin B. Laufer, Matthew B. Carroll

**Affiliations:** a Department of Internal Medicine, Keesler Medical Center, 301 Fisher Street, Biloxi, MS 39534, USA; b Department of Rheumatology, Keesler Medical Center, 301 Fisher Street, Biloxi, MS 39534, USA

**Keywords:** Hepatitis C virus, US military retiree, HCV antibody, HCV screening, Chronic HCV

## Abstract

**Background:**

In 2012, the Centers for Disease Control (CDC) recommended hepatitis C virus (HCV) screening for those born between 1945 and 1965. Prior recommendations endorsed screening based on risk factors (RFs). Because United States (US) military retirees have had at least 20 years of access to free comprehensive health care, mandatory physical fitness tests, periodic health assessments and mandatory drug screening, we hypothesized that the prevalence of HCV amongst military retirees is lower than the national average. Thus the new CDC screening guidelines may not be applicable or cost effective in this particular population.

**Methods:**

A quality improvement (QI) initiative implemented the new birth-cohort CDC screening guidelines for the internal medicine (IM) clinic of our hospital (QI group). An age-matched group from the same IM clinic, screened based on RFs for HCV infection, served as the comparator (RF group). The prevalence of the anti-HCV antibody and chronic infection was determined and compared with each other and with the national average.

**Results:**

The prevalence of the HCV antibody was 2.1% and 2.3% in the QI and RF groups, respectively (odds ratio (OR): 1.08, 95% CI: 0.37 - 3.21, P = 1.000). The prevalence of chronic infection was 0.4% and 1.8% in the QI and RF groups, respectively (OR: 4.39, 95% CI: 0.80 - 24.13, P = 0.083). When our data were compared with the national average, there were no statistical differences in the prevalence of the HCV antibody; however, there was statistically more viral clearance, and subsequently less chronic infection, in the QI group versus the national average.

**Conclusions:**

The military retiree population did not have a lower prevalence of the HCV antibody than the American populace whether screened based on age or traditional RFs. Thus, the CDC guidelines are applicable in this population. One interesting finding of this study is the higher rate of viral clearance in military retirees when compared with the national average. It is therefore possible that military retirees may be more likely to have natural viral eradication than the civilian population.

## Introduction

In 2012, the Centers for Disease Control (CDC) issued a guideline that recommended one-time birth-cohort screening for hepatitis C virus (HCV) amongst individuals born between 1945 and 1965 [1]. The rationale for this screening included the estimated high prevalence of disease in this cohort, approval of medications with improved side effect profiles and improved viral eradication, and evidence that early detection and treatment could prevent long-term sequelae of chronic infection [1]. Previous guidelines recommended that only those with risk factors (RFs) for HCV infection be screened. Traditional RFs for HCV infection are a history of injection drug use, receipt of clotting factor concentrates made before 1987, receipt of a blood transfusion or organ transplant prior to July 1992, long-term hemodialysis, persistently elevated aminotransferase levels, an HCV-positive sexual partner, needle stick injuries in health care workers, HIV/AIDS, and HCV-positive mother to child transmission [1]. The most common RF for HCV infection in the United States (US) is a history of injection drug use [2].

Patients with acute HCV will go on to develop either spontaneous viral clearance or chronic infection defined as persistent viremia. Individuals with prior HCV exposure and subsequent viral clearance are seropositive for the HCV antibody, but seronegative for HCV ribonucleic acid (RNA). Individuals with chronic infection are positive for both the HCV antibody and RNA. Rates of viral clearance vary widely, ranging from 0 to 80%, and depend on several factors including gender, ethnicity, and an acute clinical presentation of HCV [3].

Studies published over the last 15 years have demonstrated different HCV exposure rates for certain groups within the 1945 and 1965 birth cohort. While the prevalence of the HCV antibody in the general US population for those born between 1945 and 1965 is reported at 3.25% [4], the prevalence has been estimated to be between 10.3% and 15.0% for the US veteran population [5, 6]. US veterans are individuals who served in the US Armed Forces and either fulfilled their contractual service obligations or became ineligible for active service due to an acquired disability. US veterans qualify for healthcare benefits at Department of Veterans Affairs (VA) facilities. In contrast, the estimated prevalence in the US active duty population is lower, between 0.04% and 0.48% [7, 8].

US military retirees are a subset of US veterans that served at least 20 years in the armed forces. Due to their length of military service, they have had access to free comprehensive health care and mandatory screening programs that target some of the RFs for HCV infection over decades. When actively serving in the military, US military retirees underwent mandatory fitness assessments, physical fitness tests, pre- and post-deployment fitness assessments, and random drug tests which collectively increased the identification of personnel with chronic infections. For example, in 1971 routine involuntary screening of military personnel for illicit drug use was initiated by then President Nixon. US military personnel who screened positive were subsequently discharged, thus potentially eliminating from continuing military service those at a higher risk of being exposed to HCV. In 1973 the US transitioned to the all-volunteer force. This transition from a mixed conscripted and volunteer force to an all-volunteer force resulted in a larger career force with a higher percentage of married personnel and an increase in the educational level of personnel [9]. After military service, US military retirees continue to have access to free health care. Over several decades we postulate these factors have likely cultivated a lower risk group of people that comprise our current US military retiree population.

In the context of the aforementioned transformation of the US military to the all-volunteer force starting in 1973, we hypothesized that our current US military retiree population born between 1945 and 1965 would be at lower risk of exposure to HCV, thus the prevalence of chronic HCV infection would be lower than the US populace and screening therefore may not apply.

## Materials and Methods

A quality improvement (QI) project was initiated at our medical treatment facility to screen US military retirees born between 1945 and 1965 who received care in the internal medicine (IM) clinic for evidence of HCV infection in accordance with the US Preventive Services Task Force (USPSTF) recommendation statement published in September 2013 [10]. As described earlier, US military retirees are members who have served at least 20 years in the Armed Forces. Their dependents (their spouse and children under age 26) are also eligible for care and in this study data from both military retirees and dependents were gathered. Data from the QI project were collected from January 1, 2013 to April 30, 2014. This group will hereafter be referred to as the QI group. The screening test performed was the serum HCV antibody test. Once the test was performed, the result was documented in the electronic medical record as part of the subject’s health care maintenance. Positive HCV antibody tests were verified with the recombinant immunoblot assay (RIBA) or the HCV antibody verification test. Subjects with a confirmed positive HCV antibody test were then tested for the presence of chronic viremia with HCV RNA. Those with a positive HCV PCR test result were considered newly detected infections and referred for evaluation and treatment.

To determine whether or not birth cohort screening of our presumed low risk population was beneficial, we obtained HCV antibody test results to form a comparator group. This comparator group consisted of retirees and their dependents who sought care in the IM clinic during a 16-month timeframe from September 1, 2011 to December 31, 2012 (the time preceding the QI project). US military retirees and their dependents in the comparator group were all born between 1945 and 1965. They were all screened for evidence of HCV infection based only on clinical RFs and at the discretion of the physician evaluating them. Reasons given for screening for HCV infection included an elevation in transaminases, exposure to a sexually transmitted infection, report of right upper quadrant pain, or radiographic evidence of fatty liver, cirrhosis, or a liver mass. This group will hereafter be referred to as the RF group. All positive HCV antibody test results recorded during this time were further evaluated by an extensive chart review to determine if the subject had known HCV infection. Those without documentation of known HCV infection, a positive HCV antibody test, and a detectable viral load on HCV PCR testing were considered newly detected infections. If they had not already been referred for evaluation and treatment, they were subsequently referred.

Since we hypothesized that our military retiree population would have a lower prevalence of HCV exposure and chronic infection than the US populace, we compared our data to the 2003 - 2010 National Health and Nutrition Examination Survey (NHANES) data.

The study was approved by the institutional review board at our medical facility with the need for written informed consent waived. For the statistical analysis the Mann-Whitney U test was performed to assess the difference in age between the two groups. Chi-squared testing was used to assess the difference in gender, ethnicity, HCV antibody test, and HCV PCR testing between the two groups. The significance level was set to 0.05 and effect size at 0.3. For a power of at least 80%, an enrollment of 98 subjects was needed. SPSS Statistics version 21 was used.

## Results

The demographics of the QI and RF groups are shown in Table 1. The mean age was 61.6 ± 5.2 years in the QI group and 58.2 ± 6.2 years in the RF group (P < 0.001). An equal number of men and women were screened in the RF group but more women than men were screened in the QI group. There were no statistically significant differences in ethnicity noted. More retirees (as compared with dependents) were tested in the QI group as compared to the RF group, a difference that was statistically significant (P = 0.026).

**Table 1:** Demographics of the QI and RF Groups

	QI group (n = 478)	RF group (n = 221)	P value
Age ± standard deviation	61.6 ± 5.2 years	58.2 ± 6.2 years	< 0.001
Male/female	45.6%:54.4%	49.8%:50.2%	0.305
Ethnicity			
Caucasian	308 (64.4%)	130 (58.8%)	0.321
African American	69 (14.4%)	42 (19.0%)	
Asian	16 (3.3%)	5 (2.3%)	
Hispanic	1 (0.2%)	2 (0.9%)	
Other	1 (0.2%)	0	
Unknown	83 (17.4%)	42 (19.0%)	
Retiree	240 (50.2%)	131 (59.3%)	0.026
Dependent	238 (49.8%)	90 (40.7%)	

QI group: quality improvement group; RF group: risk factor group.

In the QI group, a total of 674 subjects were initially screened. There were 72 subjects who had multiple HCV antibody tests, 120 subjects born before 1945 or after 1965, and four subjects with a known diagnosis of chronic HCV, all of whom were excluded. This left 478 subjects born between 1945 and 1965 who were included in the final data analysis. Of these, 10 had positive HCV antibody test results, yielding a prevalence of 2.1% (95% CI: 1.1 - 3.8). Two of these 10 had positive HCV PCR tests, yielding a prevalence of 0.4% (95% CI: 0.1 - 1.7) who have newly detected HCV infection based on birth cohort screening. This information is presented in Table 2.

**Table 2:** HCV Antibody and PCR Positivity Rates

	QI group (n = 478)	RF group (n = 221)
HCV antibody positive test result	2.1% (95% CI: 1.1 - 3.8)	2.3% (95% CI: 1.0 - 5.2)
HCV PCR positive test result	0.4% (95% CI: 0.1 - 1.7)	1.8% (95% CI: 0.6 - 4.9)

HCV: hepatitis C virus; QI group: quality improvement group; RF group: risk factor group.

In the RF group, a total of 257 subjects were initially screened. There were 19 subjects that had a known diagnosis of chronic HCV infection and 17 subjects were screened for HCV infection by clinics other than the IM clinic. These were excluded from the final analysis. Thus 221 subjects born between 1945 and 1965 were included in the final data analysis. Of these, five subjects had positive HCV antibody test results, yielding a prevalence of 2.3% (95% CI: 1.0 - 5.2). Four of these five had detectable viral levels on HCV PCR, thus yielding a prevalence of 1.8% (95% CI: 0.6 - 4.9) who had newly detected HCV infection when screening was based on RFs and clinician discretion. This information is also presented in Table 2.

The differences between the QI and RF groups’ HCV test results are noted in Table 3. For the QI group, the prevalence of a positive HCV antibody result was 2.1%; for the RF group it was 2.3%. The odds ratio (OR) for the RF to QI group was 1.08 (95% CI: 0.37 - 3.21) (P = 1.000). For the QI group, the prevalence of a detectable viral load on HCV PCR was 0.4%; for the RF group it was 1.8%. The OR for the RF to QI group was 4.39 (95% CI: 0.80 - 24.13) (P = 0.083).

**Table 3:** Odds Ratio Results (RF to QI Group)

	OR (95% CI)	P value
HCV antibody positive test result	1.08 (0.37 - 3.21)	1.000
HCV PCR positive test result	4.39 (0.80 - 24.13)	0.083

HCV: hepatitis C virus.

Comparing our QI group alone to the 2003 - 2010 NHANES data including US non-institutionalized adults aged 20 and older who had HCV serologic testing performed (n = 20,042), the OR for having the HCV antibody was 1.10 (95% CI: 0.58 - 2.08) (P = 0.763) [11]. When a similar comparison was performed using data obtained for the RF group, no statistically significant difference was noted (data not shown). We did observe that the prevalence of chronic infection in the QI group was significantly lower when compared with the prevalence reported from NHANES between 2003 and 2010 amongst individuals born between 1945 and 1965 [11]. While the prevalence of a positive HCV RNA level in the QI group was 0.4% (95% CI: 0.1 - 1.7), the same prevalence in the NHANES group was 2.6% (95% CI: 2.1 - 3.2) [11].

## Discussion

Shaped by the complex political, social, and internal forces that transformed the US military from a combined volunteer and conscripted force to the all-volunteer force of the last few decades, our US military retirees represent a unique group of individuals who have been exposed to programs that target some of the RFs for HCV infection. With those born in 1955, the first to have been eligible to enlist in the all-volunteer force implemented in 1973, we hypothesized that our US military retirees born between 1945 and 1965 would have a significantly lower prevalence of HCV infection that the US populace. Our study demonstrated that this was not the case, that in fact US military retirees regardless of whether or not they were screened based on RFs (the RF group) or per the CDC guidelines of 2012 (the QI group) had HCV infection rates similar to those of the US populace when compared to recent NHANES data [11]. The only area where our study demonstrated a statistically significant difference were in the prevalence of chronic HCV infection as measured by PCR viral load. Here our study demonstrated that those in the QI group were more likely to clear HCV infection than the NHANES group [11].

Our study has several strengths. First, it provides evidence of how well the CDC HCV screening guidelines perform in a “real world” clinical environment like ours. Second, though there is an abundance of data regarding the prevalence of HCV in the general US population, data on the US military retiree population are limited. To our knowledge, the last study to publish information about the prevalence of HCV antibody positivity amongst US military retirees was published in 2001 [8]. Hyams et al published an HCV antibody prevalence in their military retiree population of 1.7% (95% CI: 1.2 - 2.4) [8]. While this prevalence is consistent with that observed in our study, the population they surveyed was younger than ours (average age was 44 years old) [8]. With the US military retiree population a relatively unique group within the US populace, it would appear that the prevalence has not changed over the last 10 - 15 years. Third, one of our study’s interesting observations was the higher rate of viral clearance and subsequent lower prevalence of chronic HCV infection based on detectable viral load when our QI group was compared to the NHANES data. Although Caucasian ethnicity and female gender are typically associated with greater rates of spontaneous viral clearance [3, 12], no such correlation was seen in the QI group. In fact, in the QI group about half of those with evidence of spontaneous viral clearance were middle-aged African American men, a group that is thought to be less likely to experience spontaneous viral clearance. While it is not possible to elucidate why the QI group had such a high rate of viral clearance, our study was adequately powered to have observed a true difference.

Our study does have several limitations. First, the data that we analyzed were retrospective. As with any retrospective case-control study, the effect of confounders, missing data, and inaccurate data cannot be completely accounted for and thus could potentially lead to the generation of incorrect conclusions about the population studied [13]. Additionally, retrospective studies make it difficult to establish a true cause and effect relationship [13]. Second, regarding differences in the HCV antibody positivity prevalence in the QI and RF group, our statistical analysis observed an extremely small effect size (0.01). This small effect size generated a power of 0.06, thus we were underpowered with our current sample size to detect small differences in the prevalence of HCV antibody positivity. This significantly increases the risk of reaching a false negative conclusion and thus a much larger cohort would need to be studied [14]. Third, as supported by the insufficient power for the HCV antibody prevalence between the QI and RF groups, our study reviewed data for a modest cohort of 699 subjects. Indeed, most studies measuring the prevalence of HCV antibody positivity have over 1,000 subjects. Fourth, while we sought to determine the prevalence of HCV antibody and detectable RNA level in a group of US military retirees, our population was a mix of both retirees (those who served in the military for at least 20 years) and their dependents, the latter whom most likely had no military service history. This is important to mention because these dependents would not have been subject to the same routine involuntary illicit drug screening, mandatory health examinations, or other forces which have shaped those who serve more than 20 years in the all-volunteer force.

In summary, US military retirees do not have a lower prevalence of HCV exposure than the general US population. Based on these results, the birth cohort screening strategy recommended by the CDC remains effective for our unique population. It is of interest that we detected a higher rate of spontaneous viral clearance in the QI group than the general population, but the reason for this finding is uncertain. Perhaps asymptomatic military retirees have a higher rate of spontaneous viral clearance; further studies would be required to examine this and draw conclusions. While our data are instructive, they should be viewed in the context of the limitations of the design and suboptimal power related to the HCV antibody prevalence discussed earlier. Though a prospectively designed study could provide additional insight about the prevalence of HCV antibody positivity and chronic HCV infection amongst US military retirees, the CDC guidelines note that HCV antibody testing remains highly accurate in low prevalence populations [10].
